# A Hitchhiker’s Guide to Physical and Cognitive Effort

**DOI:** 10.1007/s40279-026-02510-8

**Published:** 2026-08-07

**Authors:** Lukas Hack, Israel Halperin, Wanja Wolff

**Affiliations:** 1 Dynamics of Human Performance Regulation Laboratory, Department of Movement Science, University of Hamburg, Hamburg, Germany; 2 Department of Health Promotion, School of Public Health, Gray Faculty of Medicine and Health Sciences, Tel-Aviv University, Tel-Aviv, Israel; 3 Sylvan Adams Sport Science Institute, Tel Aviv University, Tel‑Aviv, Israel

## Abstract

Effort is integral to the regulation of behavior. It can be central for achieving goals in a range of activities, including sports, exercise, learning, and work. For centuries, researchers have tried to understand what effort is and how it shapes human behavior. Yet, despite its importance, there is no overarching consensus on what effort represents and how it should be measured. Attesting to this, academic disciplines conceptualize and measure effort in strikingly different ways. While this highlights the multitude of factors relevant to understanding effort, it has also led to siloed approaches, making interdisciplinary integration challenging. To facilitate interdisciplinary exchange, this guide aims to provide a descriptive and impartial overview of frequently used effort conceptualization and proxies. We first review three prominent conceptualizations of effort: force-based, resource-based, and mediation-based accounts. Second, we present a broad overview of commonly used effort proxies, reflecting changes in one system: neurophysiological, metabolic, psychological, and behavioral output. For each, we provide a brief review of properties, applications, and limitations relevant to effort-related research. With this approach, our aim is not to resolve disciplinary differences by pitting the utility of different conceptualizations and proxies against each other. Rather, we aim to provide a shared conceptual framework on which more integrative theorizing and cumulative progress in effort research can be achieved across disciplines.

## Key Points

**Table:** 

Effort is conceptualized and measured differently across domains. This has led to a fragmented and highly heterogeneous literature that can make domain-general integration challenging.
Effort may be best understood as a multidimensional latent construct. This necessitates integrative approaches that are explicit about their underlying theoretical assumptions and methodological choices.
No single universal marker of effort has yet been identified. Instead, effort proxies can be categorized around distinct systems (e.g., neurophysiological, metabolic, psychological, and behavioral output), each capturing a partial aspect of the construct.

## Introduction

Effort has been a *hot topic* for centuries [1–5]. Researchers have tried to provide answers to seemingly basic questions, such as, *what is effort, and how should we measure it?* This longstanding interest can be explained by the simple observation that effort is integral to the regulation of behavior (e.g., in academia [6], sports [7], learning [3], or emotion regulation [8]). Yet, despite substantial efforts to understand effort, there is no universal agreement on what the answer to any of these questions is. In fact, when referring to effort, researchers often mean different things [9–11], study it as a domain-specific concept (and not as a domain-general concept) [10], and measure it in vastly different ways [7, 12–16]. While these varied approaches have greatly advanced our context-specific knowledge of effort, they have also produced a diverse research landscape with relatively little exchange between different branches of effort research. To facilitate such exchanges more easily, we will provide an impartial descriptive interdisciplinary overview of current answers to the same old questions: *what do researchers mean by effort and what proxies do they use to quantify it?*

To provide sufficient context, we will first briefly cover contemporary effort conceptualizations. These are not mutually exclusive but rather highlight the complexity of effort and the different angles through which it can be studied. In the main part of the paper, we will then review a wide range of proxies that are used in effort research. Here, each entry briefly addresses why, how, and where a proxy is used in effort research in a question-and-answer glossary. Taken together, just as Douglas Adams’ famous *The Hitchhikers Guide to the Galaxy* helps people navigate a complex universe without panicking [17], here, we aim to do the same: help researchers and practitioners navigate the vast landscape of effort research. Thus, don’t panic. In this paper, we do not offer a new definition of effort, nor do we introduce new approaches to measure it; rather, we hope to provide a useful guide to aid researchers and practitioners in accurately situating their work within effort research. With this interdisciplinary guide, we aim to support researchers in choosing a conceptual angle and corresponding proxies that best align with their effort-related research questions. In keeping with the idea of a *hitchhiker’s guide to physical and cognitive effort research*, this paper is structured with a reader in mind who might not read it from top to bottom but regularly browses parts of it for answers to their specific effort-related questions.

## Many Answers to a Simple Question: What is Effort?

Defining effort in human sciences is complex, and multiple conceptual approaches have been proposed [10, 18]. Attesting to its relevance, researchers from a broad range of disciplines, such as cognitive psychology [19], exercise science [1], organizational psychology [20], and clinical psychology [21], have developed different conceptual approaches towards effort. The following section guides through the most common conceptualizations of effort.

### Force-Based Accounts

Force-based accounts straightforwardly conceptualize effort as the force that is required to accomplish a task [22]. More specifically, the force that is applied against a resistance is conceptualized as a vector with magnitude and direction [9]. For example, lifting a rock requires voluntary muscle contraction, and the resulting force that moves the rock against gravity would be considered effort. Here, effort intensity is defined by the amount of force required to overcome resistance [22, 23]. In contrast, involuntary muscle contractions, such as cramps, or externally generated contractions via transcranial magnetic stimulation would not be considered effort because they are not goal oriented. Force-based accounts and their associated proxies are intuitively applicable to exercise science, where physical effort is often quantified as the force that is exerted during a task [15, 24]. Beyond exercise science, advocates of force-based accounts have emphasized the concept of mental forces and their role in guiding behavior [25–27]. Everyday language terms, such as *willpower* or the *strength of will*, are frequently used to characterize the mental forces that are exerted in the service of goal-directed behavior. In turn, (mental) effort has been conceptualized as the exertion of mental forces against some other resistive forces [22]. Here, the driving force is determined by the desired goal, whereas the resistive force opposes this goal-directed action [9, 28]. For instance, consider a Stroop task with the goal to correctly respond to an incongruent trial: The goal-oriented driving force would be to categorize a color word (e.g., the word “red” presented in green font) according to its meaning, while the resistive force would be the impulse to categorize it according to its font color.

### Resource-Based Accounts

Resource-based accounts conceptualize effort in terms of the resources that are used to fuel goal-directed behavior [25], where effort intensity scales resource utilization [29]. By focusing on the energy requirements of effort-demanding actions, resource-based accounts tend to emphasize the biological basis of effort [30–33]. These accounts have been adopted by researchers across physical and cognitive domains [34, 35], and a number of candidate resources have been studied [29, 36, 37]. For example, when studying the effort of marathon running, a researcher might track limited bodily resources (e.g., glucose) that fuel the working muscles [37]; and in the cognitive domain, cardiovascular reactivity has been used as an indicator of resource utilization during tasks with varying processing demands [33]. Interestingly, resource-based accounts are not restricted to metabolic resources, and other researchers focus on structural or functional processing constraints as factors that limit cognitive and motor control processes [38–41]. Here, effort would not reflect the utilization of a limited metabolic resource but emerge as an experiential signal, indicating the costs of using one’s limited processing capacity to achieve a goal [42]. To illustrate, an incongruent Stroop trial requires participants to allocate limited cognitive resources (e.g., attention) to meet the task’s goal, which is to categorize the word’s meaning rather than its font color.

### Mediation-Based Accounts

Mediation-based accounts conceptualize effort as a mediator between two factors: (1) the characteristics of a task and the individual’s available information processing capacity, and (2) the resulting task performance [19]. Task characteristics and available capacities set the theoretical performance limits, while effort shapes the resultant performance. Returning to the Stroop example, task characteristics would be the demands of different Stroop trials (e.g., congruent and incongruent), capacity could refer to the individual’s information processing capacities, and the observed performance would be measured by response time and accuracy. Mediation-based accounts are prominent in cognitive effort research [43, 44] but also apply to physical effort [45]. Imagine a cyclist being able to ride at an average of 300 Watts for an hour (i.e., capacity). To win a race against her colleague, she needs to sustain 270 Watts for an hour (i.e., task characteristics). Here, effort mediates between her cycling capacity and the target average power output, on one hand, and the actual average power she ends up cycling over the hour.

### Effort Through Different Lenses: Interim Summary

Substantial efforts have been undertaken to structure and unify how effort is defined [9, 10], but no definition has been universally agreed upon. Yet, it can be argued that the leading definitional approaches tap into different facets of the same underlying latent construct. Force-based, resource-based, and mediation-based accounts approach effort from different angles that emphasize different aspects of effort: the forces it generates, the energetic resources it consumes, or the underlying process. Thus, the diversity of definitional approaches may be a reflection of the constructs’ inherent conceptual broadness. To benefit from these perspectives, researchers could triangulate conceptual approaches [46], to facilitate a better understanding of how effort shapes behavior before, during, and after an action [47]. However, because these accounts focus on different facets of effort, they rely on distinct assumptions that impose specific challenges for measurement:

- Force-based accounts align most intuitively with the physical domain, where effort can be defined as the force required to overcome resistance. Their application to cognitive tasks is less straightforward, as identifying and measuring the driving and resisting forces is challenging. These accounts rely on the assumption that the direction and magnitude of effort can be measured [22], but remain agnostic about what fuels the exerted force.
- Resource-based accounts focus on what fuels effort, therefore assuming that the intensity of effort can be measured through the turnover of resources [22]. While these accounts offer a clear biological grounding, identifying the resources that are specific to effort and the degree to which effort can be explained through the utilization of limited resources is still debated in the physical [25, 44, 48, 49] and cognitive domains [26, 39, 50–53] alike.
- By conceptualizing effort as a mediator between task demands, capacity, and realized performance, mediation-based accounts focus on dynamics [19]. However, meeting this assumption requires determining an individual’s true capacity. This is at least difficult, and in some cases may be impossible, as capacity can fluctuate as a function of other factors, such as learning [48, 54] or fatigue [55], complicating the accurate quantification of effort. Moreover, this account implies that effort translates directly into performance; however, this might not always hold, as effort and performance can be dissociated [56].

Addressing these challenges requires researchers to explicitly state which conceptualization they adopt and to clarify how the selected proxy aligns with this. In the next section, we review the proxies that are most frequently used across different effort conceptualizations and research domains.

## More Answers to a Simple Question: How to Measure Effort?

Effort has been measured by a plethora of methods [10], and there is no universal agreement whether a measure qualifies as an effort marker [18, 57] that captures effort itself or only relates to it via effort-adjacent phenomena [11]. To account for this heterogeneity, the proxies can be grouped according to the systems that researchers have primarily associated with effort and the output it generates: neurophysiological, metabolic, psychological, or behavioral output.^1^ Depending on one’s conceptual stance, these might not be considered direct representations of effort, but they are widely used proxies [1, 11]. While this broad categorization will not resonate with every effort researcher, the proxies discussed under these headings recur across the literature, making them essential to cover (see Table 1). To facilitate the practical utility of this overview, each proxy’s presentation centers around the same questions:

- General overview: What does the proxy assess?
- Use in effort research: In which contexts and why is it used in effort research?
- Data and methodology: How is it measured and with what temporal resolution?
- Limitations: What should be considered when using that proxy?

**Table 1:** A list of commonly used effort proxies and how they are typically used in effort research

Proxy	What it assesses?	How is it measured?	Example research use	In a nutshell
**Neurophysiological**				
Electrodermal activity	Skin conductance Sympathetic innervation of the eccrine sweat glands Physical and cognitive effort	Tonic (skin conductance level) or phasic (skin conductance response) Electrodes/wearables Continuously Microsiemens or millivolts	Measured during a Stroop task, showing larger responses in more demanding trials [13]	Sympathetic nervous system marker with high temporal resolution Sensitive to motion, temperature, and arousal Requires controlled conditions
Heart rate	Number of cardiac muscle contractions over time Cardiovascular response Physical and cognitive effort	Electrocardiography, photoplethysmography, ballistocardiography, stethoscopes, pulse palpation Continuously Beats per minute	Measured during graded treadmill tests, showing higher heart rate in higher effort levels [78]	Cardiovascular marker that can be normalized to capacity Sensitive to heat, health status, and fatigue Less established in the cognitive domain
Heart rate variability	Variability of time interval between heart beats Parasympathetic regulation Physical and cognitive effort	Electrocardiography, photoplethysmography, ballistocardiography Continuously Time or frequency domain	Measured in a working memory task, showing higher changes in more demanding trials [96]	Parasympathetic nervous system marker that allows tonic analysis Sensitive to motion, health status, and affective influence Requires controlled conditions
Pre-ejection period	Time from ventricular depolarization onset to aortic valve opening Sympathetic activation Primarily cognitive effort	Electrocardiography, impedance cardiography Continuously Milliseconds	Measured in a Flanker task, showing cardiovascular changes with higher trial demands, but no consistent pre-ejection period effects [12]	Sympathetic nervous system marker with a high temporal resolution Requires data averaging and is sensitive to arousal Requires controlled conditions
Blood pressure	Ventricular pressure driving blood into vessels Cardiovascular load Physical and cognitive effort	Invasive; intra-arterial transducers (continuously) Non-invasive; auscultatory/oscillometric (continuously); tonometry/volume clamp (momentary) Millimeters of mercury (as systolic or diastolic blood pressure)	Measured in a working memory task, showing higher systolic blood pressure values in more difficult tasks [33]	Cardiovascular marker across various domains Sensitive to health status and age Requires stationary conditions
Oxygen consumption	Exchange of oxygen intake and carbon dioxide removal Metabolic demands Almost exclusively physical effort	Spirometry Continuously Liters or milliliters	Measured in a graded physical exercise test, showing a positive association with effort levels [122]	Metabolic marker for whole-body physical effort Can be normalized to capacity, but with limited cognitive utility Requires stationary conditions
Pupillometry	Task evoked pupil size changes Neurophysiological activation Primarily cognitive effort	Eye-trackers (glasses or screen-based) Continuously Pupil dilation/constriction	Measured in a handgrip task, showing a positive relation to subjective ratings of effort and difficulty levels [135]	Autonomic nervous system marker with a high temporal resolution Sensitive to arousal, luminance, and emotion Requires controlled conditions
Hemodynamic responses	Task-evoked cerebral blood flow, oxygenation, and metabolites changes coupled to neural activation Hemodynamic responses to behavior Physical and cognitive effort	Functional near-infrared spectroscopy, functional magnetic resonance imaging, positron emission tomography Continuously and momentary Method-specific temporal and spatial metrices	Measured as blood oxygenation level-dependent changes of the ACC and the prefrontal cortex during a Stroop task, showing ACC activation in more conflict-demanding trials [143]	Neuro-metabolic index of behavior Sensitive to other behavioral and neural processes Mainly requires stationary conditions Limited temporal resolution
Electrophysiological responses	Task-evoked electrical responses in central and peripheral tissue Neural processing of behavior Physical and cognitive effort	Electroencephalography, electromyography Continuously and momentary Method specific temporal spatial metrices	Measured as event-related potentials via the electromyography in a physical decision-making task [152]	Neural index of behavior. Sensitive to artifacts and sweat production Limited spatial resolution
**Metabolic**				
Glucose	Task-related energy supply Peripheral and cortical metabolic activity Physical and cognitive effort	Invasive blood sampling (momentary) Wearable sensors (continuously) Millimoles per liter or milligrams per deciliter	Measured after a word retrieval task, showing a positive link between glucose levels and cognitive performance [161]	Metabolic marker that is well-established in physical effort Sensitive to nutrition and health status Mainly requires momentary assessment
Adenosine triphosphate	Cellular energy currency Supports muscular contraction and neural transmission Physical and cognitive effort	Functional magnetic resonance imaging, magnetic resonance spectroscopy, positron emission tomography, chromatography, mass spectrometry (continuously) Muscle biopsy (momentary) Millimolar	Measured during a knee extension task, showing increased turnover rates with increased task intensity [174]	Metabolic marker that indexes energy supply Invasive measure with limited cognitive application Requires stationary conditions
Lactate	Byproduct and signaling molecule of cellular metabolism Intensity of the mitochondrial activation Physical and cognitive effort	Invasive blood sampling (momentary) Wearable sensors (continuously) Millimoles per liter or milligrams per deciliter	Measured as post-exercise lactate kinetics after running exercises, showing to be associated with better running performance [192]	Metabolic marker that allows for normalization to capacity Strong physical base with unclear role in cognitive settings Mainly restricted to momentary analysis
**Psychological**				
Ratings of perceived exertion	Perceived exertion Psychological experience associated with a task Physical and cognitive effort	Domain-specific rating scales: Borg’s Rating of Perceived Exertion scale, NASA Task Load Index, visual analog scale (momentary) Temporal experience tracing (continuously) Discrete numbers/traces	Measured via Borg’s CR10 and visual analog scale during a submaximal physical test, showing to be positively related to higher demand levels [200]	Index for the experiential state Depends on individual’s ability and willingness to report Language and/or conceptual limits interpretation
**Behavioral output**				
Task performance	Task outcome Behavioral output Physical and cognitive effort	Sensors (e.g., force dynamometer) Experimental software Continuously/momentary Newton, reaction time, speed, accuracy, force precision	Measured as running speed in a physical test, showing to be related to ratings of perceived exertion and lactate levels [201]	Direct outcome measure with great flexibility across domains Allows for normalization to capacity Performance does not always equal the effort

This is a non-exhaustive list of commonly used effort proxies

*ACC* anterior cingulate cortex

Thus, each proxy is described as a standalone entry in our hitchhiker’s guide to physical and cognitive effort, offering a concise overview of its use. Naturally, these proxies might differ with respect to how intuitively they align with different effort conceptualizations. In keeping with the impartial approach in this hitchhiker’s guide to physical and cognitive effort, we do not position proxies as ideal or unsuitable for any given effort conceptualization, but rather aim to provide an overview that allows effort researchers to assess which proxy is most consistent with their research question and effort conceptualization.

### Neurophysiological

#### Electrodermal Activity

##### Overview

Electrodermal activity (EDA) reflects sympathetic innervation of the eccrine sweat glands of the periphery and indicates the general electrical properties of the skin [58, 59]. These sweat glands are highly sensitive to activation of the autonomic nervous system (ANS) [59, 60].

##### Use

The sympathetic nervous system (SNS) is aroused during behavior [13]; thus, EDA has long been used as a proxy of effort [13, 61, 62]. EDA has been studied across various fields including physical [63, 64], cognitive [13, 65, 66], and emotional effort [67, 68]. Here, effort intensification is expected to increase SNS activity, which can be detected by changes in EDA [69]. Consistent with this, higher EDA has been linked to greater effort perception [13, 62, 70], various indicators of cognitive effort [65, 71], and sustained physical effort [72, 73].

##### Data and Methodology

Skin conductance level and skin potential are EDA proxies. The former is measured in microsiemens (µS) and quantifies how well the skin can conduct an externally applied current. The latter is measured in millivolts (mV) and quantifies the naturally occurring electrical potential difference of the skin [74]. EDA is typically measured using finger electrodes in controlled laboratory settings to maximize internal validity. Therefore, EDA is mostly used in stationary, controlled laboratory settings with low noise and stable humidity. The continuous EDA signals vary in their time scale as they are expressed either in tonic terms or as phasic electrical responses [75]. Phasic responses reflect rapid potentials from a short-term event-related sudomotor burst, whereas tonic levels capture the slower, stimulus-independent baseline arousal [60, 70]. This allows for flexible research designs that focus on fast effort responses to a stimulus, as well as the continuous level of effort exerted over time.

##### Limitations

Although wearable EDA sensors have been developed for field-based settings [60], EDA’s sensitivity to artifacts (e.g., sweat production, motion artifacts) and the various factors that influence SNS activity (e.g., arousal) can make data interpretation challenging [76]. Measured either way, it is important to be aware of stimulus habituation that might lead to blunted EDA activity [76].

#### Heart Rate

##### Overview

Heart rate (HR) is a cardiovascular proxy that reflects the number of cardiac muscle contractions over time [74]. HR, mainly regulated by the ANS, reflects the balance between metabolic supply and demand during behavior [77–79].

##### Use

HR is frequently used to study physical effort [78, 80], due to its tight coupling with physical exertion across most of the effort spectrum [33, 69, 81]. Specifically, from low to high levels of physical effort, HR increases in an approximately linear fashion and plateaus at very high effort levels [78]. HR has also been studied in cognitive effort contexts [82], but the exact relationship between HR and cognitive effort exertion remains unclear [83].

##### Data and Methodology

Assessing HR is relatively easy and inexpensive. It is expressed in beats per minute (bpm) and measured via continuous electrocardiography, photoplethysmography [84] or ballistocardiographs [85], stethoscopes [86], and manually via pulse palpation [87]. These methods provide near real-time data and can be used in controlled laboratory and naturalistic settings, allowing for flexible research designs. Researchers across disciplines widely use this proxy as a continuous effort readout [78, 88]. This flexibility distinguishes HR from some other psychophysiological proxies of effort.

##### Limitations

Although cardiac innervation is a central part of the ANS, there is no linear relationship between the sympathetic and parasympathetic nervous systems’ contributions to changes in HR [89]. This makes it challenging to interpret it straightforwardly as a proxy for cognitive effort, especially if one is interested in the exact role of the ANS in effortful behavior [34]. HR is also sensitive to external (e.g., heat) [90] and psychophysiological factors like fatigue [91].

#### Heart Rate Variability

##### Overview

Heart rate variability (HRV) reflects the changes in the interval between two successive heart beats [74]. HRV, studied for over a century [92], primarily reflects vagally mediated parasympathetic regulation of cardiac activity [93].

##### Use

HRV has been used to study cognitive effort [70, 94–97], physical effort [98–100], and clinical effort-related questions [101, 102]. Here, researchers aim to learn more about parasympathetic regulation during effortful behavior. Lower HRV generally indicates reduced parasympathetic and increased sympathetic activation, often interpreted as greater effort utilization. For example, many authors propose an inverse relationship between HRV and physical effort [98, 99] or cognitive effort [96, 103]. Other studies have reported a positive relationship between HRV and effort [66].

##### Data and Methodology

HRV can be measured in controlled laboratory settings and in the field [66]. Researchers commonly use electrocardiography to continuously obtain the raw cardiac signal [93], where raw cardiac data are averaged and processed to compute frequency or time-domain metrics [93, 104]. Besides this, other methods, including photoplethysmography, ballistocardiographs, inter-beat intervals (referred to as RR-intervals), or heart sound signals [93, 104], can be used as well.

##### Limitations

Challenges in HRV measurement pertain to motion artifacts, electrode placement sensitivity, or data processing issues (e.g., R peak detection errors) [104]. Additionally, concerns about intra- and intersubject reliability [96] are particularly present in free-moving settings and where high temporal (e.g., 80 Hz sampling rate) resolution is required to understand effort. Furthermore, across domains, the association between effort exertion and HRV dynamics is a contested topic [70, 94, 97], potentially due to HRV’s close association with non-effort-related (e.g., affective) states [93, 97].

#### Pre-ejection Period

##### Overview

The cardiac pre-ejection period (PEP) refers to the time interval between the onset of the ventricular depolarization and the opening of the aortic valve that precedes blood ejection [74]. PEP primarily reflects beta-adrenergic (sympathetic) influence on myocardial tissue [105].

##### Use

PEP is widely used in cognitive effort research [12, 33, 105, 106], and to a lesser extent in physical effort [107, 108]. Due to its inverse relationship with the SNS’s activation [74], researchers generally expect shorter time intervals between cardiac depolarization and blood ejection during effortful behavior. Indeed, numerous studies across disciplines have found shorter PEP when individuals exert effort [105].

##### Data and Methodology

Assessing PEP is relatively inexpensive, but reliable measurement typically requires controlled laboratory conditions. Since PEP is not directly observable, it requires a combination of a non-invasive continuous electrocardiography recording and impedance cardiography [12]. This ensures the estimation of the exact interval in milliseconds between the ventricular depolarization and the actual ejection of blood. Because the raw signal is averaged over time to reduce noise, PEP provides an event-related rather than continuous readout of effort.

##### Limitations

Assessing the PEP requires a high-quality signal acquisition, which can be technically demanding. Furthermore, PEP is sensitive to other arousal states, such as affective responses, which may confound effort measurements [109].

#### Blood Pressure

##### Overview

Ventricular pressure drives blood from the heart into blood vessels. This is referred to as blood pressure (BP) [110]. Researchers commonly distinguish between systolic blood pressure (SBP), the maximum pressure at the peak of an ejection, and diastolic blood pressure (DBP), the minimum pressure measured between heartbeats [111]. BP changes provide information on the central and peripheral functions due to the complex interplay between central ANS activation and peripheral vascular resistance changes [74].

##### Use

In effort research, SBP is of particular interest because it primarily reflects sympathetic myocardial activity [34]. SBP is most commonly used in physical effort research [112–115], and to a lesser degree in cognitive effort research [33, 88, 116]. In both domains, SBP generally increases with effort intensity [32, 34, 114]. Mechanistically, increased SBP reflects heightened metabolic demands of the working organs [117].

##### Data and Methodology

BP can be assessed invasively and non-invasively and is typically reported in millimeters of mercury (mmHg), as SBP/DBP [110]. Invasive approaches, such as intra-arterial pressure transducers, provide continuous data and are considered the clinical gold standard [118]. In contrast, non-invasive methods are easier to apply and more frequently used in effort research. These encompass moment-to-moment approaches (auscultatory, oscillometric), as well as continuous techniques (arterial tonometry, volume clamp) [74].

##### Limitations

BP is influenced by other factors, such as health status or age [119], complicating its interpretation as a specific proxy for effort. Additionally, non-invasive moment-to-moment measures may systematically overestimate BP during hypotension, while they tend to underestimate BP during hypertension [118].

#### Oxygen Consumption

##### Overview

The respiratory gas system facilitates the exchange of central metabolites (i.e., oxygen or carbon dioxide) between the environment and the individual. Here, the core processes are oxygen intake via inhalation and carbon dioxide removal via exhalation [74, 120]. Oxygen consumption (VO_2_) measures the volume of oxygen inhaled over a fixed time interval [121].

##### Use

VO_2_ serves as an index for the peripheral metabolic activation of the body and the central ANS regulation [74]. It is primarily studied in the context of physical effort during whole-body efforts [122–124]. This is because VO_2_ does not substantially change during single-joint physical efforts or during cognitive effort utilization. Typically, VO_2_ increases linearly with physical effort. At very high effort levels, VO_2_ and effort demands decouple, as the body's capacity limit for VO_2_ uptake (VO_2max_) has been reached [122, 123, 125].

##### Data and Methodology

VO_2_ is measured continuously via spirometry (in liters or milliliters) [121] and normalized to bodyweight. Spirometry is widely accepted as the gold standard for measuring VO_2_ during physical efforts [126].

##### Limitations

The measurement of VO_2_ via spirometry is relatively costly, mostly restricted to stationary laboratory settings, and requires careful calibration [123].

#### Pupillometry

##### Overview

Pupillometry studies pupil changes in response to a stimulus [127], with these changes serving as a readout of underlying neurophysiological processes [128]. Pupil responses are typically classified into three types: light reflex, near fixation, and psychosensory responses [129]. The latter refers to task-evoked changes in pupil size, such as dilation or constriction, which occur in response to task demands [130].

##### Use

Pupillometry is primarily used in cognitive effort research [131–134], with emerging work on physical effort [127, 135]. A large body of research points to a positive relationship between effort intensity and pupil dilation across domains [14, 38, 127, 135, 136]. These changes are thought to be linked to activity in the locus coeruleus, a brainstem area that is associated with effort and attention (for reviews, see [136, 137]).

##### Data and Methodology

Eye trackers provide a continuous readout of the pupil responses with high temporal resolution [14, 138]. Systems range from eye-tracking glasses to near-infrared screen-based devices [138, 139]. The variety of eye-tracking devices affords high flexibility in research designs.

##### Limitations

Measuring pupil changes is complex. Pupil changes are sensitive to non-effort processes, such as arousal or emotions [128, 140], as well as to changes in the external environment, such as luminance, humidity, or temperature [136]. Experimental settings that control luminance and that use auditory stimuli can offset these challenges to a degree. Also, eye-tracking devices that are capable of high-resolution pupillometry (with sampling rates around 1000 Hz) tend to be relatively costly. Furthermore, implementing pupillometry in highly flexible experimental designs can be challenging because participants need to remain in a somewhat stationary position to ensure data quality. In addition, experimental design must carefully control factors such as baseline measurement and stimulus luminance [141] to ensure data quality.

#### Hemodynamic Responses

##### Overview

Hemodynamic responses refer to the dynamic changes of cerebral blood flow, oxygenation, and metabolites coupled to neural activation in brain areas associated with effort [142].

##### Use

A substantial body of research has investigated how the brain processes effort [42, 143, 144]. For cognitive effort, a large body of research points towards the role of structures such as the anterior cingulate cortex (ACC) and the dorsolateral prefrontal cortex (dlPFC) (for reviews, see [145, 146]). In physical effort research, cortical regions have been the primary research focus, as subcortical activity is difficult to assess during physical effort with current methods. Here, dlPFC activation has often been reported as well [16, 142]. More broadly, researchers point toward common circuitries that govern physical and cognitive effort [147–149].

##### Data and Methodology

Various imaging methods have been used to study the brain’s hemodynamic response to effortful actions. The most commonly used methods are functional near-infrared spectroscopy (fNIRS) [142], functional magnetic resonance imaging (fMRI), and positron emission tomography (PET) (for a review, see [145]). fNIRS and fMRI applications allow for regional and network analysis in either block- or event-related designs. Additionally, fNIRS allows for flexible research designs, as it is not limited to stationary settings [142].

##### Limitations

Interpreting hemodynamic responses as a specific proxy for effort remains challenging, as brain regions might be active in the absence of effort, and the validity of region-to-construct mapping in the brain has been questioned more generally [146]. Moreover, these techniques involve high to very high acquisition and maintenance costs. Also, most current measures have either limited spatial (e.g., fNIRS) or temporal resolution (e.g., fMRI) and require extended training for data acquisition, processing, and analysis. In addition, susceptibility to motion artifacts can restrict their application to naturalistic research questions, particularly in the context of physical effort [142].

#### Electrophysiological Responses

##### Overview

Actions are based on neural activity. Electrophysiological responses refer to electrical activity in cortical and peripheral nervous system structures associated with effort [150].

##### Use

Researchers are commonly interested in how the nervous system guides actions [151]. For cognitive effort, researchers have investigated central nervous structures, such as the ACC and dorsomedial and dorsolateral prefrontal cortex, that may be linked to effort [151]. In the physical domain, the focus is either on how central nervous structures (e.g., motor areas) process movements or how the neural signals are transmitted and executed in the periphery [150, 152].

##### Data and Methodology

Neural activity is measured using a range of electrophysiological recording techniques. The most prominent method is electroencephalography (EEG), which allows for event-related analysis like movement-related cortical potentials (MRCPs) [150] or cost–benefit analyses [151]. Electromyography (EMG) is used to investigate neural transmission and motor execution in the periphery [152].

##### Limitations

Most electrophysiological measures have limited spatial resolution, especially when recorded at a surface level [150]. Moreover, research settings are challenging, as these measures are sensitive to motion and sweat artifacts [152], particularly in the context of physical effort.

### Metabolic

#### Glucose

##### Overview

Glucose is a metabolic resource with fluctuating availability based on task duration and intensity, as well as nutrient intake [153]. It is the primary cellular energy source of peripheral (plasma glucose) and cortical (brain glucose) activity [29, 154–156].

##### Use

The association of glucose with effort—primarily in the physical domain—has been studied for over a century [37, 157, 158]. Human glucose regulation depends on glucose delivery, transport and metabolism [159, 160]. For example, in mild to moderate physical tasks (30–60% of VO_2max_), glucose delivery towards the muscle cell increases until a plateau is reached. Here, higher levels of effort are associated with increased delivery, provided that adequate glucose is available [158]. In the cognitive domain, findings on glucose dynamics are unclear [156]. While some propose a link between cognitive effort and brain-glucose usage [25, 29, 161], evidence is mixed [50, 162, 163]. However, neuroimaging studies suggest an increase in blood oxygenation level-dependent (BOLD) signals—an index for metabolic activation—in respective brain areas during cognitively demanding tasks [164, 165].

##### Data and Methodology

Glucose can be assessed in the field or in controlled laboratory settings via glucose tracking after glucose administration or deprivation—for example, after consuming a glucose-rich drink or after restricting food intake. Glucose dynamics are then tracked using invasive or non-invasive measurement devices [156, 166]. In humans, peripheral glucose concentration is measured via invasive handheld blood glucose assessment devices [156]. Here, the instantaneous level of blood glucose (in mg/dL or mmol/L) is measured, usually prior to and after a task [167], to analyze glucose consumption. Non-invasive continuous measures are also gaining popularity [166, 168].

##### Limitations

The reliability of non-invasive measures has been questioned [166, 169]. Despite higher reliability using invasive methods, in particular, brain glucose is difficult to assess in vivo [25]. Also, glucose is central for many core processes; that is why glucose levels also change in various non-effort-related states, like sleeping [170].

#### Adenosine Triphosphate

##### Overview

Adenosine triphosphate (ATP) is a high-energy molecule that stores and delivers cellular energy [171]. Additionally, it provides energy for neurotransmission in the sympathetic and parasympathetic nerve branches [171, 172].

##### Use

ATP indicates cell metabolism and energy supply for the working organ. It is mainly studied in the context of physical effort, such as the intensity of a muscle contraction [1, 173]. Here, the temporal dynamics of ATP utilization depend on the duration and the level of intensity. Importantly, ATP stored in the working muscle can support muscle activation for only up to a few seconds of work [174]. With an increase in muscle activation, the level of ATP breakdown increases up until the ATP demands exceed the resynthesis capacity and other metabolites are used for the continuation of effort exertion [171, 173, 175, 176]. Besides its application in physical contexts, has also gained popularity in cognitive effort research [29, 177]. Here, ATP utilization seems to scale dynamically with cognitive and sensory demands. For example, ATP production in task-relevant cortical regions is associated with an increase in task demands [29].

##### Data and Methodology

ATP is typically measured using imaging techniques, such as PET or fMRI, or chemo-physical methods like liquid chromatography or mass spectrometry [177]. These measures provide a quantitative readout of regional cerebral ATP levels in millimolar (mM) units at rest and during work [178]. In physical effort, besides local imaging methods [175], muscle biopsy samples, as an invasive measurement, are used to detect changes in the ATP concentration of human tissue [173]. Imaging techniques restrict studies to rather stationary settings, affording less flexibility in research designs.

##### Limitations

Tracking ATP during effort is a challenge because currently available measurement technologies are not well suited to capture nuanced continuous fluctuations related to effort over time. Moreover, it requires expensive approaches and has relatively low spatiotemporal resolution [177].

#### Lactate

##### Overview

Lactate is a key metabolite in cellular energy metabolism, where it serves as both an energy substrate and a signaling molecule [179, 180]. It is produced and utilized across various tissues such as muscles, brain, and red blood cells [181]. Lactate levels are sensitive to internal factors (e.g., health status) [182] and external factors such as task demands [1, 183].

##### Use

Lactate changes during exertion reflect the intensity of mitochondrial activation and the process between production and uptake. Traditionally studied in physical effort contexts [36, 184], a large body of research has established strong but non-linear coupling between lactate concentration and physical effort demands [183]. Physical exertion triggers a rise in lactate production, which plateaus under sufficient oxygen supply. When task demands exceed aerobic capacity (e.g., > 60% VO_2max_), lactate accumulates exponentially [36, 183, 185]. Although its role in cognitive effort is less clear [181], lactate is increasingly being studied in relation to cognitive effort dynamics [186] and clinical conditions [182]. Here, lactate has been studied as a signaling molecule that is involved in cerebral processes such as memory and neuronal excitability [187–190].

##### Data and Methodology

Lactate concentration, typically expressed in millimoles per liter (mmol/L) or milligrams per deciliter (mg/dL), is most commonly assessed via invasive blood sampling at the fingertip or the earlobe [191, 192]. These methods provide only discrete, task-dependent moment-to-moment insights into lactate kinetics. Interestingly, wearable microneedle sensors have been developed recently [193–196], promising the continuous assessment of lactate [179, 197].

##### Limitations

Despite promising methods to measure lactate with a higher temporal resolution, continuous measures are mainly applied in clinical contexts, and their reliability for research purposes has not yet been verified [198]. Moreover, lactate does not only accumulate in response to effort but also due to non-effort-related processes, such as chronic inflammation and cancer [199].

### Psychological

#### Ratings of Perceived Exertion

##### Overview

Exerting effort is typically accompanied by a distinct subjective sensation of it. This perception of effort (PE) refers to the experiential dimension of effort [7, 202].

##### Use

Researchers and practitioners are often interested in how humans perceive exertion and how this shapes the behavior [7, 47, 203]. To study PE, researchers commonly rely on ratings of perceived exertion. These ratings are widely used in both physical and cognitive domains [7, 204, 205]. Robust evidence supports that when effort demands increase, subjective ratings of perceived exertion tend to increase as well [200, 204, 206, 207]. Importantly, even under constant external demands (e.g., resistance on a bike ergometer), self-reported ratings can change, indicating that how effortful something feels depends on how (long) a person has interacted with a task [13].

##### Data and Methodology

PE is typically assessed with domain-specific subjective rating scales administered during or immediately after a task [1]. Such proxies assume that humans can reliably report their interoceptive experience related to an action [10, 204, 206]. In physical effort, variants of Borg's Rating of Perceived Exertion (RPE) scale (i.e., CR10, Borg 6–20) are the most prominent such proxies [7, 204], which are easy to administer, due to verbal anchors [208]. In cognitive effort, the NASA Task Load Index (NASA-TLX) [205] and visual analog scales [206, 207, 209] are commonly used, and offer more fine-grained data, as they rely on precise input. More recently, proxies such as temporal experience tracing (TET) have been used to retrospectively assess PE with a higher temporal resolution [210–213].

##### Limitations

Some researchers question the validty of PE ratings, as they depend on introspective abilities and individuals’ willingness to provide accurate reports [214]. This limitation is particularly relevant for populations with reduced abilities to do so [215, 216]. Additionally, linguistic or conceptual misunderstandings can compromise data quality [209]. Also, participants may differ in how they interpret the scales, depending on the anchors provided to them [217], the verbal instructions they receive [208], and the situational context [15, 203]. Furthermore, younger populations may interpret the visual analog scale in a logarithmic rather than a linear fashion [18]. Importantly, other (psychological) sensations, such as fatigue or boredom, may be even more closely related to neurophysiological changes than PE itself [13, 218]. Thus, subjective ratings may reflect a mixture of changes in different systems linked to effort.

### Behavioral Output

#### Task Performance

##### Overview

Task performance reflects (at least partly) the output of effortful behavior [19, 219]. Performance can be captured in terms of magnitude, duration, or precision of an outcome [220, 221], and it can be studied overtly, covertly [222], or by unpacking underlying performance processes [223].

##### Use

While effort and performance are thought to be closely linked [1], the exact relationship may be influenced by several factors (for an overview, see [56]). Nonetheless, performance is widely assessed in effort research, sometimes even as a stand-in for effort itself, because it captures how invested effort translates into outcomes across domains [224]. In principle, any outcome that effort is directed at can be used as a proxy of task performance. In the physical domain, one of the most common ways to quantify effort via tasks is using force outputs [15, 203, 225]. Force is the magnitude of output produced by a working organ (e.g., the quadriceps) against a resisting load [9]. Forces can be measured dynamically or isometrically against maximal (e.g., 1 repetition maximum [1RM] or maximal voluntary contractions) and submaximal (e.g., time to task failure or accuracy of force production or motor efficiency) resistance. In the cognitive domains, magnitude-based measures (e.g., response times) [19] precision-based measures (e.g., error rates) [226], mediation-based measures (i.e., drift diffusion modeling) [223], and duration-based measures (e.g., time to failure) [227] are the most frequently used types of performance proxies. For this, researchers often rely on standardized, computer-based response-conflict tasks, like Stroop [131] or Flanker [228], or on vigilance tasks, such as the Sustained Attention to Response Task [229].

##### Data and Methodology

Task performance can be recorded in near real time with high temporal precision, particularly in controlled lab settings, but increasingly in naturalistic contexts too. Performance outcomes (e.g., reaction time, speed, force) are often logged automatically by experimental software or dedicated sensors (e.g., Newtons or kilograms using handgrip dynamometers) [15, 135], whereas other outcomes can require post-processing, for example, to assess precision or error rates [220, 221]. In principle, an infinite number of task performance proxies can be conceived, thereby making them highly flexible.

##### Limitations

The raw assessment of task performance as an effort proxy relies on the assumption that the exerted efforts translate directly into performance outputs. However, this assumption must not always be true, as various phenomena (e.g., fatigue, strategy) may corrupt this direct translation [56]. Accordingly, this places high demands on researchers to tailor their performance proxy in the best way to their research question.

## Conclusion

A one-size-fits-all effort proxy might not exist (or has not yet been universally agreed upon), and the degree of its conceptual and empirical delineation from associated constructs like PE and task performance is an ongoing riddle for effort research. To reduce the risk of circular explanations [230], where the explanation of a pattern is already the phenomenon (e.g., effort), researchers should justify their conceptual and methodological choice before the data collection process. Complicating matters further, different operationalizations vary in their temporal and spatial resolution [231], and their relationship with one another differs across different effort intensities [15, 50]. As a result, each proxy often only provides a temporally and spatially restricted glimpse into effort and the regulatory mechanisms that underlie it [1]. For example, continuous proxies such as pupillometry or force production provide high-resolution insights into the temporal dynamics of effort, while snapshot proxies like lactate or self-reported ratings capture broader or discrete changes. This increases the risk of drawing over-simplified or over-generalized conclusions when interpretations are only based on one effort proxy. Furthermore, many effort proxies are sensitive to non-effort phenomena (e.g., arousal, temperature), which further complicates their interpretation. To account for this, researchers might consider triangulating different effort proxies to reflect the diversity of theoretical and methodological angles [46]. In the spirit of network analytical approaches—in contrast with more reductionist one-to-one mapping approaches [232]—effort research might benefit from combining proxies that mirror the various systems through which effort emerges (e.g., neurophysiological, metabolic, psychological, behavioral output). Importantly, each conceptualization and system imposes specific measurement challenges. For example, studying effort through a force-based perspective requires measuring the produced output against a resisting force. In contrast, when studying effort through a mediation-based perspective, one should track a participant’s maximum capacity to truly tap into the process of effort as a mediator between capacity and performance. This becomes more complicated because the participant's momentary maximum capacity is likely to dynamically change as the task unfolds. Importantly, the same effort proxy can be informative for different effort definitions. Measuring force may be aligned with the mediation-based account when assessed as relative force to a person’s maximum force capacity. However, it may also be aligned with the force-based account if measured as raw physical force output. Together, this highlights the importance of being explicit about the features that are most relevant to answer one’s research question, and to align concept and proxy based on them. In this hitchhiker’s guide to physical and cognitive effort, we aimed to highlight the complexity of the effort universe. This complexity is rooted in the compound nature of any effortful behavior and the challenges associated with studying it. As we have demonstrated, researchers employ various conceptualizations and operationalizations, each providing distinct insights into different aspects of effort. This diversity can be a limitation if left implicit or a strength if researchers clearly state which conceptualization they adopt and how their chosen proxy aligns with it. Our aim was to provide an impartial map of conceptual and methodological options, making it easier to navigate this diversity and thereby advancing understanding of the old hot topic: effort.

## Footnotes

1 An open question is whether subjective and objective effort should be treated as distinct latent constructs, and, if so, what their hierarchical relationship might be. Likewise, the association between effort and performance is complex and depends on which (subjective or objective) effort and performance proxies are analyzed [56]. Addressing these questions is beyond the scope of this paper and will require dedicated research. However, we deem it important to point out that different perspectives on these questions might be useful for different research questions. For example, psychologists are often interested in how subjective effort shapes behavior and how objective proxies feed into this experience. In contrast, sport scientists may be more focused on identifying physiological substrates that are specific to effort and distinguishing them from performance outcomes.
